# Supplementary figures and images for: Inter-slice leakage and intra-slice aliasing in simultaneous multi-slice echo-planar images
Source: Brain Struct Funct. 2020 Mar 5;225(3):1153–8. doi: 10.1007/s00429-020-02053-2 (PMC7166208; doi:10.1007/s00429-020-02053-2)

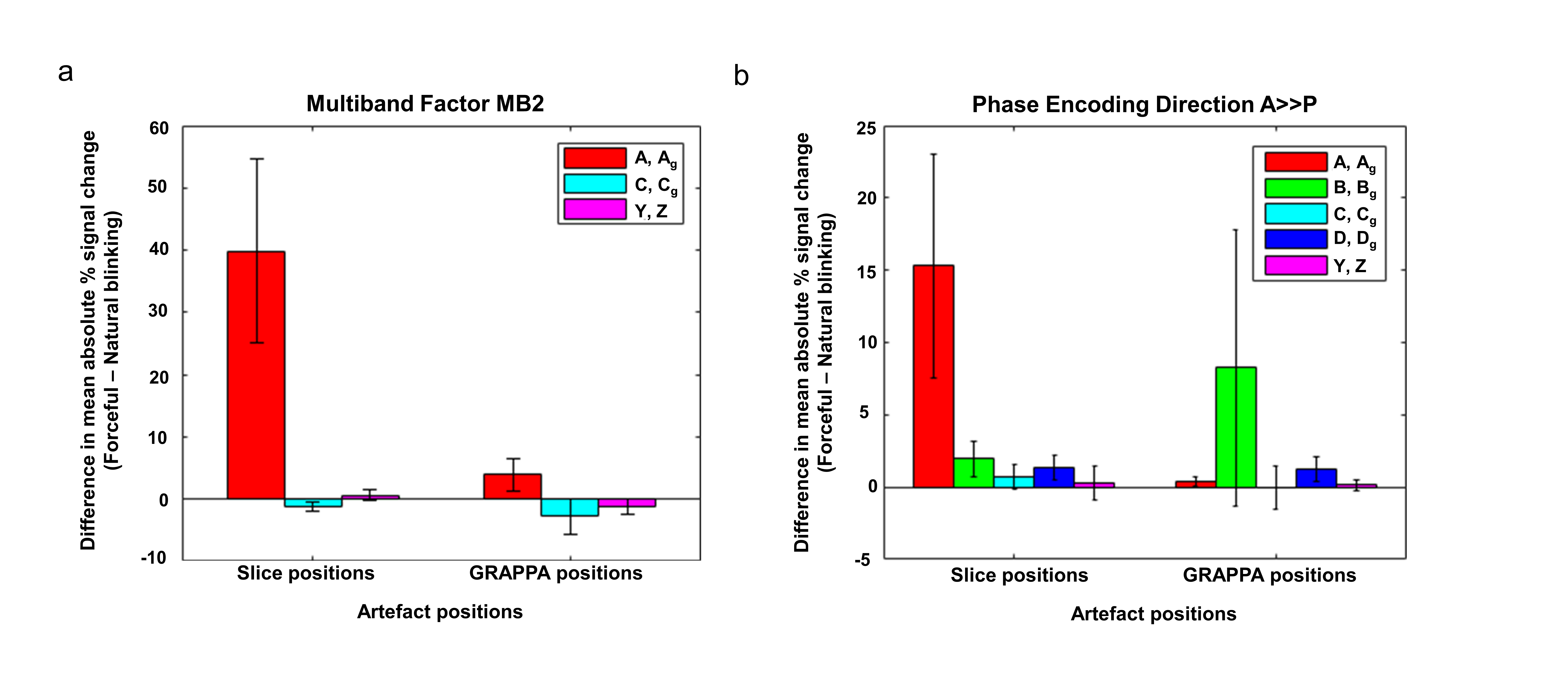

Supplement: Supplementary file 1 — Supplementary file1 (TIF 2338 kb) [file 429_2020_2053_MOESM1_ESM.tif]

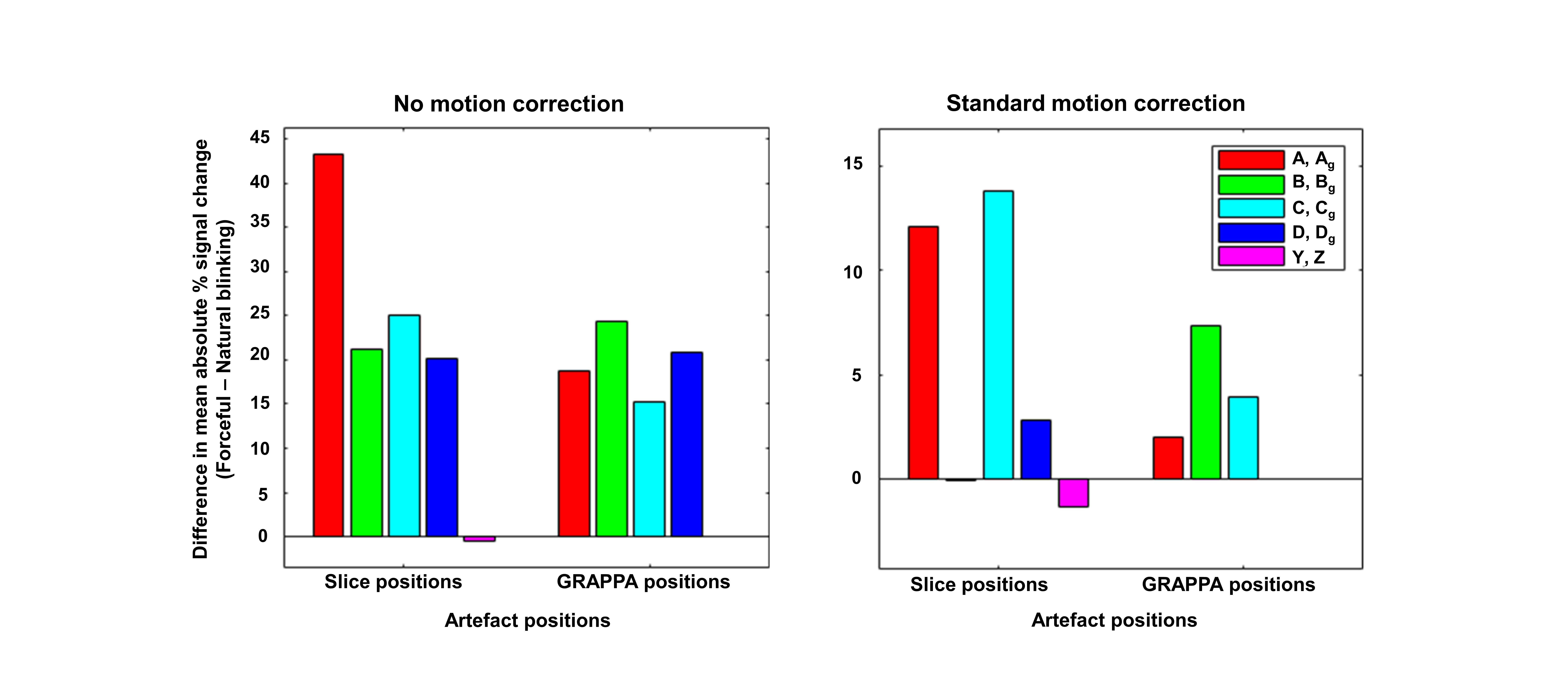

Supplement: Supplementary file 2 — Supplementary file2 (TIF 2892 kb) [file 429_2020_2053_MOESM2_ESM.tif]

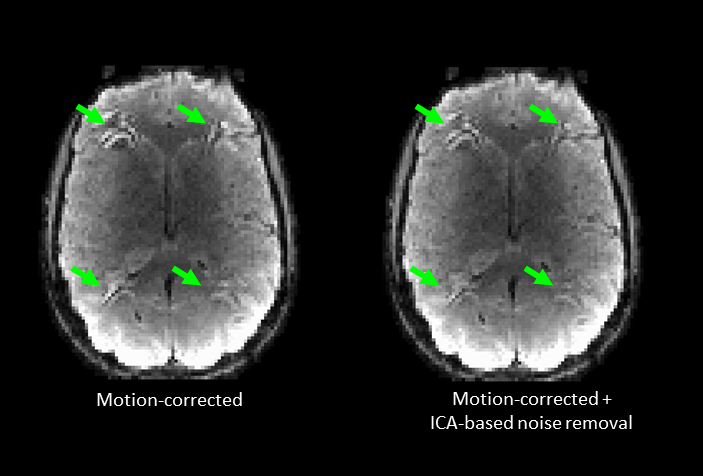

Supplement: Supplementary file 3 — Supplementary file3 (TIF 225 kb) [file 429_2020_2053_MOESM3_ESM.tif]

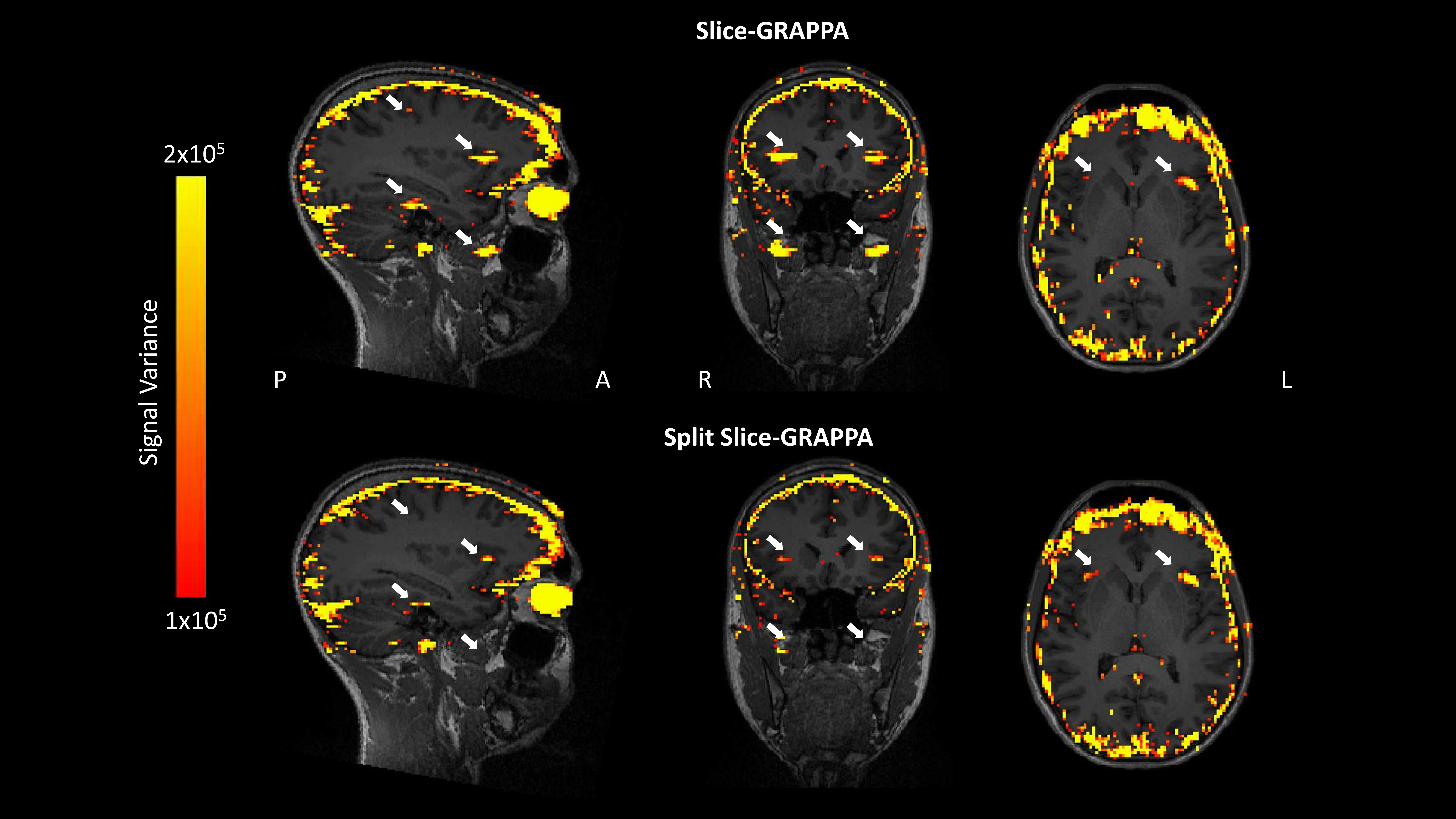

Supplement: Supplementary file 4 — Supplementary file4 (TIF 13629 kb) [file 429_2020_2053_MOESM4_ESM.tif]
